# Prevalence of active trachoma and its associated factors among 1–9 years of age children from model and non-model kebeles in Dangila district, northwest Ethiopia

**DOI:** 10.1371/journal.pone.0268441

**Published:** 2022-06-15

**Authors:** Almaw Genet, Zewdu Dagnew, Gashaw Melkie, Awoke Keleb, Achenef Motbainor, Amare Mebrat, Cheru Tesema Leshargie

**Affiliations:** 1 Awi Zone, Dangila Woreda Health Office, Dangila, Ethiopia; 2 Department of Public Health, College of Health Sciences, Debre Markos University, Debre Markos, Ethiopia; 3 Department of Environmental Health, School of Public Health, Teda Health Science College, Gondar, Ethiopia; 4 Department of Environmental Health, College of Medicine and Health Sciences, Wollo University, Dessie, Ethiopia; 5 School of Public Health, College of Medicine and Health Sciences, Bahir Dar University, Bahir Dar, Ethiopia; 6 School of Public Health, College of Medicine and Health Science, Wolldia University, Woldia, Ethiopia; Yenepoya Medical College, Yenepoya University, INDIA

## Abstract

**Background:**

Trachoma is the leading infectious disease that leads to blindness worldwide, especially in developing countries. Though Ethiopia had targeted a trachoma elimination program by 2020, the problem worsens, particularly in the Amhara Region. Even though sustained intervention measures are undertaken across the region, it is unclear why trachoma is still a significant public health problem. So, this study assessed the prevalence of active trachoma and associated factors among 1–9 years of age children from model and non-model kebeles in Dangila district Amhara Region, Northwest Ethiopia.

**Methods:**

A community-based comparative cross-sectional study was conducted from 20^th^ September 2019 to 29^th^ October 2019. A multistage stratified random sampling technique was used to reach 704 children from model and non-model kebeles. Samples were allocated proportionally to model and non-model kebeles. A structured and pretested data collection tool and observational checklist was used to manage the necessary data. Data were coded and entered in Epidata version 4.6, and further analysis was done using SPSS version 20 software. Bivariable and multivariable logistic regression analysis was employed to identify factors associated with active trachoma. Adjusted Odds Ratios (AOR), p-value, and respected Confidence Interval (CI) were used to report the findings.

**Results:**

Seven hundred four children were included in this study, with a response rate of 97.8%. The overall prevalence of active trachoma was 6% (95% CI: 4.5, 8.1). The prevalence of active trachoma among non-model and model Kebele was not significantly different. Still, the prevalence of active trachoma among children from model Kebele were [4.5%, (95% CI: 2.4%, 7.1%)] relatively lower compared with non-model kebeles, [7.6%, 95% CI: (4.9%, 10.9%)]. Moreover, not using latrine (AOR = 4.29, 95% CI: 1.96, 9.34), fly-eye contact (AOR = 2.59, 95% CI: 1.11, 6.03), presence of sleep in eyes (AOR = 2.46, 95% CI: 1.10, 5.47), presence of ocular discharge (AOR = 2.79, 95% CI: 1.30, 6.00), presence of nasal discharges (AOR = 2.67, 95% CI: 1.21, 5.90) and washing faces with soap (AOR = 0.22, 95% CI: 0.07, 0.69) were found significantly associated with the prevalence of active trachoma among children 1–9 years old.

**Conclusions:**

The prevalence of active trachoma in the model and non-model kebeles was high and did not show a statistical difference. Attention to be given to latrine utilization, washing face with soap, and other personal hygiene activities.

## Introduction

Trachoma is a disease of the eye caused by infection with the bacteria Chlamydia Trachomatis [1, 2]. Blindness from trachoma is an irreversible and endemic disease in 44 countries worldwide [2]. Active trachoma causes follicular inflammation of the tarsal conjunctiva. Children under nine years are the primary reservoir of the bacteria for active trachoma because active trachoma prevalence decreases while aging increases [3, 4]. Model kebeles were set by the Ethiopian government’s five-year health sector strategic transformation plan to combat infectious diseases, specially NTDS, including active trachoma [5]. A model kebele needs to be free from open defecation, adequately constructed, utilized latrine, and improved personal and environmental hygiene. Furthermore, improved school health where the residents meet the following interrelated criteria as ≥80% of residents are model households, ≥85% of residents attended facility-based delivery, ≥85% of residents owned improved latrines, and ≥85% of schools become models [5].

Globally, around 50 million people are infected with trachoma, and 3 to 10 million people are becoming blind due to the infection [6]. The annual economic loss due to trachoma-related blindness and visual impairment was 2.9–5.3 billion US dollars [6, 7]. Nowadays, 40.6 million people worldwide suffer from active trachoma, of which 77% are from 29 endemic African countries, including Ethiopia [8]. Ethiopia, India, Nigeria, Sudan, and Guinea, account for 48.5% of the global burden of active trachoma [1]. In Ethiopia, trachoma is the second major cause of blindness and the third major cause of low vision [9]. Despite the sustained effort that excelled in stopping the problem, trachoma is still a significant public health problem in Ethiopia, with the peak prevalence in Amhara Region at 62.6% [10–12].

Several factors contribute to the occurrence of active trachoma. Evidence identified that secretions around the eyes that attract flies, scarcity of water for personal hygiene, limited access and utilization of latrines, overcrowded living conditions, close contact, and sleeping in the same bed with infected persons increased trachoma prevalence [13–16]. The occurrence of active trachoma among 1–9 years of age-old children was also associated with different socio-demographic characteristics like age, family size, sex status [16–18], educational level of the household head, and household economic status [17, 18].

SAFE (Surgery, Antibiotics, Facial cleanliness, and Environmental improvement)-based interventions have been implemented in endemic countries, including Ethiopia [19, 20]. Due to that, the Dangila district graduated as free of trachoma four years ago by achieving < 5% prevalence of active trachoma and stopping Mass Drug Administrations (MDA). Therefore, the only intervention left for sustaining a < 5% prevalence of active trachoma is the F and E components of the SAFE strategy by implementing health extension packages that substantially affect maintaining lower trachoma prevalence in the district. However, evidence on the effect of health extension packages on the prevalence of trachoma after stopping MDA in the study area is limited [21]. Therefore, considering the problem and variability of risk factors, this study aimed to assess the prevalence of active trachoma and associated factors among children aged 1–9 years old in the health extension package non-model and model kebeles of Dangila District in Ethiopia.

## Methods

### Study design, setting, and period

A comparative cross-sectional study was conducted in Dangila district from October to November 2019. Dangila district is the 3^rd^ most populous and covers a large geographical area among the twelve districts at Awi-zonal administration. Danglia district (woreda) is a part of the Amhara region and Awi zone and is located in the western part of Ethiopia. Furthermore, it is found 78 km away from Bahir Dar regional city in the Southeast direction. The district had six health centers and thirty-one health posts or kebeles. Of these, 15 kebeles were verified as model kebele on the district transformation agenda by model kebele criteria. The rest 16 are non-model kebeles [21]. The district’s projected total population based on the 2007 national population census in 2020 is 154,876 and 35,786 households [22].

### Study population

All children whose age was found in the range of 1–9 years old in the Dangila district were the source population. Children aged 1–9 years living in the model and non-model kebeles were included in this study. On the contrary, temporarily residing children were excluded.

### Study variables

The outcome variable showed signs of active trachoma among children aged 1 to 9 years old. The explanatory variables were age and sex of the child, residence, parental education, wealth index, availability of latrine facility, type of latrine, latrine utilization, household size, source of water, accessibility of water, the quantity of daily water used, presence of feces near the compound, waste disposal sites, solid waste disposal methods, availability of livestock, frequency of face washing, using soap for washing, discharge on the eye, facial cleanness and presence of flies on a child’s face, knowledge of mothers about trachoma and number of under ten years children.

### Operational definitions

**Active trachoma: The** presence of Trachomatous inflammation–follicular or Trachomatous inflammation- intense [6].

**Trachomatous inflammation -follicular (TF):** The presence of five or more follicles in the upper tarsal conjunctiva, each with at least 0.5 mm diameter in size [23].

**Trachomatous inflammation- intense (TI):-** is pronounced inflammatory thickening of the upper tarsal conjunctiva that obscures more than half of the standard deep vessels [23].

**Knowledge**: assessed by developing trachoma transmission, prevention and control measures related questions. If the mother answered ≥ 80% of knowledge-related questions, then the mother classified as knowledgeable 50%-79% was reasonably knowledgeable. Less than 50% were classified as less knowledgeable [24].

**Model kebeles:** where the residents meet the following interrelated criteria as ≥80% of residents are model households, ≥85% of residents attended facility-based delivery, ≥85% of residents owned improved latrines, and ≥85% of schools become models [5].

**Non-model kebeles:—**The low performance of the five interrelated criteria, namely; model HH (≤80%), Facility delivery (≤85%), improved latrine ownership (≤85%), and model schools (≤85%)as well as for urban kebeles it includes model youth centers performance (≤85) [5].

### Sample size determination and sampling procedures

The sample size was determined by using the second objective using factors significantly associated with active trachoma by considering the following assumptions: 95% confidence level, 80% power of the study, two comparison groups population ratio 1:1, considering the availability of flies on the face children as significant determinants of trachoma and the proportion in the exposed group was 45.9%, the proportion in the unexposed group was 29% with an adjusted odds ratio (AOR) of 1.98 and design effect 2 [25]. The calculated sample size was 720 households with 1–9 years of age-old children, including a 10% non-response rate.

Multistage stratified sampling followed by a simple random sampling technique was used to select kebeles and households. Stratification of the kebeles was done based on their model and non-model kebele status. Of all kebeles in the district, 35% (5 kebeles in the model and five kebeles in the non-model) were randomly selected. Those kebeles are Chara-01, Dubi, Dimsa, and Affessa from graduated model kebeles; on the other way, Gumdri, Abadra, Misrak Zelesa, Gissa Balegiziabher, and Dengeshita were selected randomly from non-model kebele strata. Population proportion allocation was proposed to determine the required sample size for each randomly selected kebele.

In a total of 2087 and 3049 households, at least one children were aged 1–9 years old in model and non-model kebele, respectively. Households with children aged 1–9 years old were selected using a systematic random sampling technique. To determine the interval of Households in selected Kebele, the K^th^ interval value was used. Every 8^th^ and 5^th^ interval was used to allocate the sample size to model and non-model kebeles, respectively. Before starting the sampling, pen spiring was conducted to indicate the starting location of the village. Finally, one child was selected using the lottery method when two or more children live in 1–9 years old per a single household. Finally, 720 households were selected using a systematic sampling technique.

### Data collection tools and procedures

Data were collected using a pretested structured questionnaire, observational checklists, and physical examination. The data collection tool was developed after reviewing other available literature [8, 15, 24, 26–30]. The questionnaire had five parts: socio-demographic variables, child behavioural factors, parents or caregivers’ knowledge, and environmental or household-related variables. Firstly, the questionnaire was developed in English, translated into Amharic (local language), and returned to English to check its consistency. Four trained public health professionals who collected the household data had previous data collection experience. The clinical assessment of active trachoma signs in each child was assessed by two TT-trained surgeon nurses using 2.5x loupes. The data collector documented the presence of any ocular or nasal secretions and any fly-eye contact during data collection before clinical examination of children’s eyes. Trained TT surgeon trachoma grader nurses examined both eyes for active trachoma signs using the WHO Trachoma Grading System [29]. The examiner cleaned his hands with a disinfectant solution (alcohol) before making eye contact and examining the children.

### Data management and analysis

The questionnaire was pretested using 5% of the total sample in another kebele which was not included in the study, and the necessary amendments were done after the pre-test.

After the data collection, the information was reviewed, and incomplete questionnaires were returned to the data collectors for revisiting the respondent and completion. Data were coded and entered in Epidata version 4.6 and exported to SPSS version 20 for analysis. Descriptive statistics were used to describe data. Principal Component Analysis(PCA) generated a wealth index and divided households into poor, medium, and high economic groups.

The sampling adequacy for PCA was assessed using Kaiser-Meyer-Olkin (KMO) statistics. The KMO measures the adequacy of sampling thoroughly and the sampling adequacy for each variable that indicates the proportion of variance the variables might be caused. Sampling adequacy set at communality value > 0.5 with P-value > 0.05 and complex structure factor (Eugene value) greater than one was considered [26]. Bivariate and multivariable logistic regression analyses identified variables significantly associated with active trachoma. Model fitness was checked using the Hosmer-Lemeshow test (P-value < 0.05). A backward stepwise logistic regression model was used during multivariable logistic regression to control confounding effects. During bivariable logistic regression analysis, variables with a p-value < 0.25 were considered candidates for multivariable logistic regression analysis [27, 30]. Statistically significant association of independent variable with dependent variable was declared at P-value < 0.05.

### Ethical considerations

Debre Markos University assessed ethics. An approval letter was obtained from the College of Health Sciences Ethical Review Committee. Moreover, the support letter was secured from the study setting, Dangila district Administrative and Health offices. The parents or guardians were informed about the study’s purpose, and verbal informed consent was obtained before data collection. Mothers or caregivers were also informed that they had the full right to discontinue participating in the study. Confidentiality was ensured by omitting the name and ID of the participants. A separated, secured, and conducive interview setting was selected. Each respondent was assured that their information was confidential and used only for research. Trachoma-infected children were referred to the nearest health facility.

## Results

### Socio-demographic characteristics of the respondent

A total of 704 children in the age range of 1 to 9 years completed their responses, providing a response rate of 97.8%. Slightly more than half (55%) of the respondents were from the model kebeles. The mean (± standard deviation (SD)) age of children was 4.8 (±2.3) years and 4.0 (±2.2) years old from model and non-model kebeles, respectively. The proportion of female children who participated in this study was 185 (52.6%) from the model and 182 (51.7%) from non-model kebeles. The majority, 347 (98.6%) of the households, have two children, and 345 (98%) of them were from model and non-model kebeles. Regarding household economic status, 140 (39.8%) of households in the model and 95 (27%) were in the high financial group based on assets owned (Table 1).

**Table 1:** The respondents’ socio-economic and demographic characteristics from model and non-model kebele of Dangila district, northwest Ethiopia, 2019 (n = 704).

Characteristics		Kebele category				Totally		Chi-square
		Model Kebele (n = 352)		Non-model kebele(n = 352)				
		No	%	No	%	No	%	P-value
Sex	Male	167	47.4	170	48.3	337	47.9	0.763
	Female	185	52.6	182	51.7	367	52.1	
Education status of a child	Not attended	246	69.9	281	79.8	527	74.8	0.002
	Primary	106	30.1	71	20.1	177	25.2	
Education status of mothers	No formal education	265	75.3	292	83	557	79.1	0.085
	Primary & above	87	24.7	60	17	147	20.9	
The educational level of husbands	No formal education	203	64.3	254	75	457	69.8	0.025
	Primary and above	113	35.7	85	25	198	30.2	
Children’s age	1–5 years	215	61.1	261	74.1	476	67.6	0.001
	6–9 years	137	38.9	91	25.9	228	32.4	
Age of mothers or caregivers	≤ 29	50	14.2	107	30.4	157	22.3	0.001
	30–44	251	71.3	223	63.4	474	67.3	
	45–59	51	14.5	22	6.2	72	10.4	
Family size	≤ 5	220	62.5	169	48	389	55.3	0.001
	>5	132	37.5	183	52	315	44.7	
Sleeping in the same bed	Yes	142	42	265	45.4	407	57.8	0.001
	No	210	58	87	54.5	297	42.2	
Wealth index	Poor	113	32.1	120	34.1	233	33.1	0.001
	Medium	99	28.1	137	38.9	236	33.5	
	Higher or rich	140	39.8	95	27	235	33.4	
Residence	Urban	71	20.2	12	3.4	83	11.8	0.001
	Rural	281	79.8	340	96.6	621	88.2	

### Environmental and housing conditions of the households

Nearly all households had sanitation facilities in their compound. For instance, 97.8% and 96% of model and non-model kebele households had latrine access. Of them, 98.6% from model kebele and 72.7% from non-model kebele properly utilize latrine. However, human feces were detected near 3.7% and 50.8% of households among model and non-model kebeles, respectively (Table **2**).

**Table 2:** The households’ environmental and housing conditions in the model and non-model kebeles in Dangila district, northwest Ethiopia, 2019 (n = 704).

Characteristics		Model Kebele (n = 352)		Non-model kebele (n = 352)		Total		Chi-square
		No	%	No	%	No	%	P-value
Have latrine	Yes	344	97.8	338	96	682	96.9	0.192
	No	8	2.3	14	4	22	3.1	
Use latrine (n = 682)	Yes	347	98.6	240	72.7	587	86.1	0.001
	No	5	1.4	90	27.3	95	13.9	
Presence of solid waste disposal pit	Yes	228	64.8	195	55.4	423	60.1	0.011
	No	124	35.2	157	44.6	281	39.9	
Water source	Improved	336	95.5	317	90.1	653	93	0.006
	Unimproved	16	4.5	35	9.9	51	7	
Water source distance	in the yard	54	15.3	11	3.1	65	9.2	
	<30 minute	262	74.4	295	83.8	557	79.1	0.001
	> = 30 minute	36	10.2	46	13.1	82	11.6	
Presence of liquid waste	Yes	239	68	156	44	395	56.1	0.001
	No	113	32	196	56	309	43.9	
human faces near the house	Yes	13	3.7	179	50.8	192	27.3	0.001
	No	339	96.3	173	49.2	512	72.7	

### Childhood behavioral factors

Of 704 children, 669 (95%) had a habit of face washing. In contrast, only fifteen 15 (4.3%) in model and 20 (5.6%) in non-model kebeles had no tradition of washing their face regularly. There is a difference between children’s model and non-model kebeles’ hygiene behavior, especially facial hygiene. Similarly, 55.4%, 19.9%, and 29.7% of the children from the model kebele were observed washing their faces once, twice and more than twice a day, respectively, using soap. On the contrary, 73.5%, 17.5%, and 9% of the children from the non-model kebele were presented with washing their faces once, twice and more than twice a day, respectively, using soap (Table **3**).

**Table 3:** Childhood behavioral factors in the model and non-model kebeles in Dangila district, northwest Ethiopia, 2019 (n = 704).

Characteristics		Model Kebele (n = 352)		Non-model kebele (n = 352)		Total		Chi-square
		No	%	No	%	No	%	P-value
Face washing regularly	Yes	337	95.7	332	94.3	669	95	0.386
	No	15	4.3	20	5.6	35	5	
How many times wash your face per day?	One	170	55.4	244	73.5	414	61.8	
	Twice	67	19.9	58	17.5	125	18.6	0.001
	> twice	100	29.7	30	9	130	19.4	
Ocular discharge	Yes	130	36.9	91	25.8	221	31.4	0.038
	No	222	63.1	261	74.2	483	68.6	
Nasal discharge	Yes	114	32.4	142	40.3	256	36.4	0.028
	No	238	67.6	210	59.7	418	63.6	
Fly- eye contact	Yes	144	40.9	186	52.8	330	46.9	0.001
	No	208	50.1	166	47.2	374	63.1	
Drying material use	Regularly	9	2.6	7	2.1	16	2.4	
	Sometimes	89	26.4	87	26.2	176	26.3	0.923
	Never	239	71	238	71.7	477	71.3	
Flies observed on the face	Yes	114	32.4	142	40.3	256	36.4	0.201
	No	238	67.6	210	59.7	448	63.6	
Use soap for face washing	Yes	343	97.4	330	94	673	95.6	0.017
	No	9	2.6	22	6	31	4.4	
Sleep in the eye	Yes	148	42	162	45.4	310	43.8	0.288
	No	204	58	190	54.5	394	56.2	
Azithromycin Rx status	None	237	67.3	283	80.4	520	73.9	0.001
	At least one time	115	32.7	69	19.6	184	26.1	

### Prevalence of active trachoma

**Fig 1:**
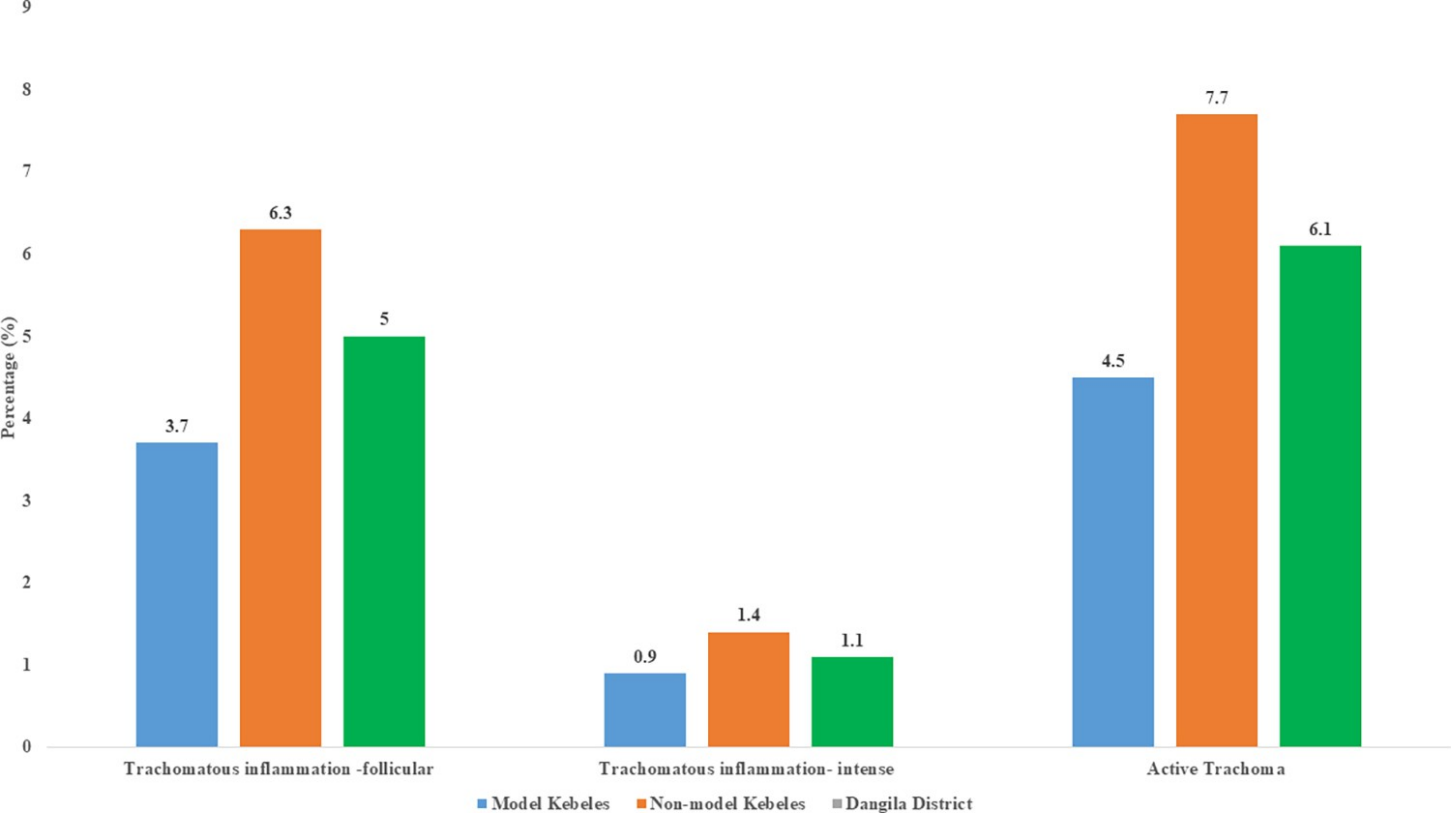
Prevalence of active trachoma among children aged 1–9 years from model and non-model kebele in Dangila district, northwest Ethiopia, 2019.

Of all 704 children examined for the presence or absence of trachoma in their eyes, 43 children were positive for active trachoma. The overall prevalence of active trachoma was 6.1% (95% CI: 4.5%, 8.1%). The prevalence found to be 4.5% (95% CI: 2.4%, 7.1%) in the model and 7.7% (95% CI: 4.9%, 10.9%) in the non-model kebeles (Fig 1).

### Factors associated with active trachoma

During the bivariate logistic regression, latrine utilization, residence, animal waste, solid waste disposal pit, ocular discharge, nasal discharge, flies on the face, fly-eye contact, sleep on the eye, antibiotics medication, and washing faces by soap were found to be a p-value less than or equal to 0.25 and selected as a candidate for the final multivariable analysis. But, the other variables, such as socio-economic variables with a p-value > 0.25, are not selected for multivariable analysis. During the multivariable logistic regression analysis, variables like the experience of fly-eye contact, the presence of sleep in the eye, the presence of discharge on the eye, face washing with soap, and nasal discharge were significantly associated with the prevalence of active trachoma.

The odds of having active trachoma among children (1–9) from households who did not utilize latrine was 4.3 times higher than children from families using latrine (AOR = 4.3, 95% CI: 2.0, 9.3). In the same manner, children who had fly-eye contact were three times (AOR = 2.6, 95% CI: 1.1, 6.0) higher than children who had no fly-eye contact. Having an ocular and nasal discharge were 2.8 and 2.7 times higher to develop active trachoma than their counterparts, as well as children’s who had a sleep (collection of crusted secretions in the canthus of the eyes) were 2.5 times more likely to develop active trachoma. Lastly, the odds of having active trachoma among children (1–9) years old who had an experience of face washing with soap were 77.9% lower than their counterparts (AOR = 0.2, 95% CI: 0.1, 0.7) (Table 4).

**Table 4:** Regression analysis of associated factors of active trachoma among children aged (1–9 years) from Dangila district, northwest Ethiopia, 2019.

Characteristics	Active trachoma (n = 704)				
	Present	Absent		COR (95%CL)	AOR (95%CL)
**Maternal education**					
Not formal Edu	37	520		0.60 (0.25, 1.45)	
Primary and more	6	141		1	
**Children living place**					
Non-model kebele	27 (7.7%)	325 (92.3%)		1.75 (0.93, 3.30)	1.23 (0.96, 2.34)
Model kebele	16 (4.5%)	336 (95.5%)		1	1
**Utilization of latrine (n = 682)**					
No	17 (17.9%)	78 (82.1%)		6.18 (3.10, 12.30)	4.29 (1.96, 9.34)**
Yes	20 (3.4%)	567 (96.6%)		1	1
**Residence**					
Urban	2 (2.4%)	81 (97.6)		2.86 (0.68, 12.06)	3.74 (0.72, 19.37)
Rural	41 (6.6%)	580 (93.4%)		1	1
**Animal faces**					
Yes	34 (8.6%)	361 (91.4%)		2.41 (1.13, 5.11)	2.07 (0.80, 5.37)
No	9 (3.8%)	230 (96.2%)		1	1
**Solid waste disposal pit**					
Yes	17 (4%)	406 (96)		1	1
No	26 (9.3)	255 (90.7)		2.45 (1.30, 4.58)	2.06 (0.95, 4.48)
**Ocular discharge**					
Yes	28 (6%)	206 (94%)		4.12 (2.16, 7.89)	2.79 (1.30, 6.00)**
No	15 (12%)	455 (88%)		1	1
**Nasal discharge**					
Yes	29(11.3)	227 (89.7)		3.96 (2.05, 7.65)	2.67(1.21, 5.90)*
No	14(3.1)	434(96.9)		1	1
**Flies on face**					
Yes	28(11.9)	206(88.1)		4.12 (2.16, 7.89)	1.99 (0.83, 4.74)
No	15(3.1)	455(96.9)		1	1
**Fly-eye contact**					
Yes	32(9.7%)	298(90.3%)		3.54 (1.76, 7.15)	2.59 (1.11, 6.03)*
No	11(2.9%)	363(97.1%)		1	1
**Sleep on the eye**					
Yes	29 (9.4%)	281(90.6%)		2.80 (1.45, 5.40)	2.46 (1.10, 5.47)*
No	14 (3.6%)	380 (96.4%)		1	1
**Antibiotics medication**					
None	3 (1.6%)	181(98.4%)		0.20 (0.06, 0.65)	0.30 (0.09, 1.06)
At least one time	40 (7.7%)	480 (92.3%)		1	1
**Washing faces with soap**					
**No**	8 (25.2%	23 (74.2%)		0.158 (0.07, 0.38)	0.22 (0.07, 0.69)**
**Yes**	35 (5.2%.)	638 (94.8%)		1	1

Signifcant at P < 0.05

*P<0.01

**P<0.001.

## Discussion

In Ethiopia, trachoma is the leading infectious disease that causes blindness and death [1]. The current study found that the overall prevalence of active trachoma infection among children 1–9 years old was 6.1% (95% CI: 4.5, 8.1%). The current study found comparable evidence with a previous study conducted in the Dera district [10], Gondar Zuria District [31], North and South Wollo Zones [32]. Furthermore, our study finding is lower than the previous findings reported in Zala district [33], Leku town [17], Medawulabo [34], and Lemo district [35]. The lower prevalence in the current study might be due to the better implementation of the SAFE strategy, such as A (antibiotics), F (facial cleanness), and E (environmental management) components. The previous study in Zala district, Leku town, and Medawulabo reported that about 85.6%, 59.0%, and 44.1% of children wash their faces regularly, respectively. However, in the current study, 95% of children washed their faces regularly every day. Moreover, the discrepancy might be because the current study setting graduated from MDA four years ago. Furthermore, environmental improvements in access to sanitation facilities and improved water sources have an essential role in reducing active trachoma in the current study. However, the finding was higher than in previous studies conducted in the Casamance region of Senegal and Amhara region, Ethiopia, after implementing a three-year SAFE strategy [36, 37].

Furthermore, the prevalence of active trachoma in non-model and model and kebele differs at 7.7% (95% CI: 4.9, 10.9) and 4.5% (95% CI: 2.4, 7.1), respectively, the difference was not statistically significant. This consistent finding might be because the two study settings have had the same awareness and knowledge about trachoma signs, transmission mechanisms, and prevention methods. About 99.4% of mothers who live in the model had information about trachoma, while approximately the exact figure (98.9%) of mothers residing in non-model kebele had information about trachoma. Similarly, 38.9% and 32.9% of children’s mothers who live in the model and non-model households had good knowledge about trachoma. Besides this, the availability of latrines in both kebeles is nearly similar. Moreover, the similarity may be due to children’s hygiene practices such as face washing and soap use in both kebeles.

The difference in model and non-model kebeles did not persist after multivariable analysis, indicating that both areas require continued implementation of trachoma control interventions to increase the prevalence under the elimination target of 5%. The lower-level maternal education might also explain the difference. The educational status of mothers has played a significant role in reducing trachoma prevalence as educated mothers had positive attention in caring for and teaching their children about trachoma prevention mechanisms. On the other hand, it may be due to the study conducted at the district discontinued MDA supplementation four years ago.

After completing five annual MDA rounds, the pooled prevalence was higher than the 5% criteria set under the WHO trachoma elimination program and impact surveys in five districts in the Amhara region [38, 39]. This similarity may be due to the implementation of SAFE interventions that results may help the district sustain a lower prevalence of active trachoma.

The pooled prevalence was higher than the 5% criteria set under the WHO trachoma elimination program; the prevalence of active trachoma in non-model kebele was higher [40]. These might be due to the re-emergence of Chlamydia infection as the district discontinued MDA supplementation (the last rounds of MDA were conducted in the district four years later in 2015). Such re-emergence in the communities is supported by previous studies conducted in five districts in Ethiopia and reported that active infection re-emergence was 1.4% in recently treated communities and 4.3% not treated communities [37].

In this study analysis, different factors have an association with active trachoma. Latrine utilization, washing faces with soap, fly-eye contact experience, sleep in the eyes, discharge on the eye and nose were variables found to be significantly associated with the prevalence of active trachoma among children (1-9y rears) old. The probability of being infected with active trachoma was higher in children 1–9 years old who had ocular and nasal discharges than those who had not. The finding is consistent with previous studies in Gambia and Tanzania [41], southern and northern Wollo zone districts, and rural Ethiopia [32, 42]. This might be due to the infected discharge from the nose and eye transmitting infection via fingers, flies, or fomites [43, 44] and through direct contact with nasal and ocular secretions [45].

This study also revealed that children (1–9) who had fly-eye contact were more likely to risk active trachoma than their counterparts. This finding is supported by the previous studies conducted in Ankober district and rural Ethiopia [42, 46]. This may be due to sleep (collection of crusted secretions in the canthus of the eyes) that is caused by due to secretion of ocular and nasal discharge in the eye. This may be due to sleep in the eye and ocular and nasal discharge. The presence of nasal and ocular secretions and crusts, which attract flies, leads to increased fly-eye contact and an increased risk of trachoma infection [28, 33, 47, 48].

Not using a latrine can be considered an indicator of hygienic behavior. Children (1–9 years old) who lived in households that did not use latrines were more likely to be affected by active trachoma. This finding aligns with a study conducted in the Gonji kolela, Baso-Liben, and Ankober districts[46, 47, 49]. This could be due to practicing open defecation near the house being a favorable environment for breeding Musca sorbents; these are a reservoir of the causative agent, Chlamydia trachomatis, and an essential contributor to disease transmission.

This study also revealed that using soap for face washing is significantly associated with reducing active trachoma prevalence. This finding is consistent with the previous studies in Leku town and the Baso-Liben district [17, 47]. The possible explanation for this may be that face washing with water and soap improves the facial cleanliness of the children, and their faces should not receive eye-seeking flies, which reduces the risk of acquiring trachoma.

## Conclusions

In this study, the prevalence of active trachoma among children 1–9 years old was 6% and had not shown statistically significant variation between model and non-model kebeles. The prevalence of active trachoma among children 1–9 years old in the Dangila District was in line with the WHO-recommended trachoma elimination program. Latrine utilization, using soap, the experience of fly-eye contact, the presence of sleep, discharge on the eye and the nose were significantly associated with the prevalence of active trachoma among children (1–9 years old). Therefore, an intervention area needs to be improving personal hygiene-related activities such as washing children’s faces thoroughly with soap, removing dirt (ocular and nasal discharge), and eye sleeping from their faces. Significant emphasis on the use of sanitation services, especially on latrine construction and use, is also required to eliminate open defecation.

## Limitation of the study

One of the strengths of this study is including large sample size. Moreover, this study investigated children in the 1–9 year age group in accordance with WHO guidelines. It is possible that the responses to the questionnaire might have social desirability bias and potential recall bias. However, many components such as environmental hygiene conditions, water source, and facial hygiene were directly observed. The other limitation of this study was the lack of qualitative components for triangulation.

## Supporting information

S1 File (DOCX)

S2 File (DOCX)

S1 Dataset (SAV)

## Acronyms/Abbreviations

AOR: Adjusted odds ratio
AT: Active trachoma
MOH: Ministry of Health
NGO: Non-Governmental Organization
NMK: Non-Model Kebeles
SAFE: Surgery, Antibiotics, Facial Cleanliness, and Environmental Improvement
TF: Trachomatis Inflammation Follicular
TI: Trachomatous Inflammation Intense
WHO: World Health Organization
CI: Confidence Interval
COR: Crude Odds Ratio
SD: Standard Deviation
SSA: Sub Saharan Africa

## References

[1] Mariotti SP, Pascolini D, Rose-Nussbaumer J: Trachoma: global magnitude of a preventable cause of blindness. *British Journal of Ophthalmology* 2009(5):563–568. doi: 10.1136/bjo.2008.148494 19098034

[2] World Health Organization (WHO): Trachoma—World Health Organization Key facts. 9 May 2021.

[3] Chawicha K, Asnake M, Kassie G, Nigatu T, Belachew M, Zerihun H: The status of hygiene and sanitation practice among rural model families of the Health Extension Program (HEP) in Wolayta and Kembata Tembaro Zones of Southern Nations, Nationalities and Peoples’ Region of Ethiopia. *Ethiopian Journal of Health Development* 2012, 26(2):93–100.

[4] Australia C: CDNA National Guidelines for the Public Health Management of Trachoma. Do Health, Editor. 2014.

[5] Federal Ministry of health: Woreda Transformation Agenda Concepts and its Theory of Change. 2018.

[6] World Health Organization (WHO): Trachoma fact-sheets (Epidemiology and clinic features, Distribution, Economic impact, Prevention and control and WHO response. 2022, 13(5):855.

[7] Resnikoff S, Pascolini D, Mariotti SP, Pokharel GP: Global magnitude of visual impairment caused by uncorrected refractive errors in 2004. *Bulletin of the World Health Organization* 2008, 86:63–70. doi: 10.2471/blt.07.041210 18235892 PMC2647357

[8] World Health Organization (WHO): Alliance for the Global Elimination of Blinding Trachoma by the year 2020: Progress report on elimination of trachoma. *Weekly Epidemiological Record = Relieve epidemiological hebdomadal* 2014, 89(39):421–428. 25275153

[9] Berhane Y, Worku A, Bejiga A, Adamu L, Alemayehu W, Bedri A, et al: Prevalence and causes of blindness and low vision in Ethiopia. *Ethiopian Journal of Health Development* 2007, 21(3):204–210.

[10] Alemayehu Metadel, Koye Digsu N, Tariku Amare, Kedir Yimam: Prevalence of active trachoma and its associated factors among rural and urban children in Dera Woreda, Northwest Ethiopia: a comparative cross-sectional study. Biomed research international 2015. doi: 10.1155/2015/570898 25954753 PMC4390108

[11] World Health Organization: Global WHO alliance for the elimination of blinding trachoma by 2020. Weekly Epidemiological Record = Relevé épidémiologique hebdomadaire 2012, 87(17):161–168. 22574352

[12] Berhane Y, Worku A, Bejiga A, Adamu L, Alemayehu W, Bedri A, et al: Prevalence of trachoma in Ethiopia. *The Ethiopian Journal of Health Development (EJHD)* 2007, 21(3).

[13] Bailey R, Downes B, Downes R, Mabey D: Trachoma and water use; a case control study in a Gambian village. *Transactions of the Royal Society of Tropical Medicine and Hygiene* 1991, 85(6):824–828. doi: 10.1016/0035-9203(91)90470-j 1801366

[14] Brechner RJ, West S, Lynch M: Trachoma and flies: individual vs environmental risk factors. *Archives of Ophthalmology* 1992, 110(5):687–689. doi: 10.1001/archopht.1992.01080170109035 1580846

[15] Oswald WE, Stewart AE, Kramer MR, Endeshaw T, Zerihun M, Melak B, et al: Active trachoma and community use of sanitation, Ethiopia. *Bulletin of the World Health Organization* 2017, 95(4):250. doi: 10.2471/BLT.16.177758 28479620 PMC5407250

[16] West SK, Congdon N, Katala S, Mele L: Facial cleanliness and risk of trachoma in families. *Archives of Ophthalmology* 1991, 109(6):855–857. doi: 10.1001/archopht.1991.01080060119038 2043075

[17] Abebo Teshome Abuka, Tesfaye Dawit Jember: Prevalence and distribution of active trachoma among children 1–9 years old at Leku town, southern Ethiopia. *Current Pediatric Research* 2017, 21(3).

[18] Assefa N, Abrham Roba A, Abdosh T, Kemal J, Demissie E: Prevalence and Factors Associated with Trachoma among Primary School Children in Harari Region, Eastern Ethiopia. *Ophthalmology Research*: *An International Journal* 2017, 7(3).

[19] World Health Organization: Global elimination of blinding trachoma. Geneva: World Health Organization; 1998. *World Health Assembly*, *Resolution* 2015, 51(1).

[20] Roba AA, Wondimu A, Patel D, Zondervan M: Effects of intervention with the SAFE strategy on trachoma across Ethiopia. *Journal of Epidemiology & Community Health* 2011, 65(7):626–631. doi: 10.1136/jech.2009.094763 20693489

[21] Dangila woreda Health Office: 2018/2019 Annual report the woreda 2019.

[22] Knoema: Population and Households Statistics of Ethiopia 17 January 2019.

[23] Australia CDN: CDNA National Guidelines for the Public Health Management of Trachoma. *Do Health*, *Editor* 2014.

[24] Lewetegn Moges, Getachew Meron, Kebede Tadesse, Tadesse Gemechu, Tsegahun Asfaw: Prevalence of Intestinal Parasites among Preschool Children and Maternal Knowledge, Attitude and Practice on Prevention and Control of Intestinal Parasites in Senbete and Bete Towns, North Shoa, Ethiopia. *International Journal of Biomedical Materials Research* 2019, 7(1):1–7.

[25] Mpyet C, Lass BD, Yahaya HB, Solomon AW: Prevalence of and risk factors for trachoma in Kano state, Nigeria. *PLoS One* 2012, 7(7):e40421. doi: 10.1371/journal.pone.0040421 22792311 PMC3391244

[26] Ayuni NW, Sari IG: Analysis of factors that influencing the interest of Bali State Polytechnic’s students in entrepreneurship. In: *Journal of Physics*: *Conference Series*: 2018: IOP Publishing; 2018: 012071.

[27] Bendel RB, Afifi AA: Trachoma and water use; a case control study in a Gambian village *Journal of the American Statistical association* 1991, 85(6):824–828. 10.1016/0035-9203(91)90470-j 1801366

[28] Mpyet C, Lass BD, Yahaya HB, Solomon AW: Prevalence and risk factors for trachoma in Kano state, Nigeria. *PLoS One* 2012, 7(7):e40421. doi: 10.1371/journal.pone.0040421 22792311 PMC3391244

[29] Thylefors B, Dawson CR, Jones BR, West SK, Taylor HR: A simple system for the assessment of trachoma and its complications. *Bulletin of the World Health Organization* 1987, 65(4):477. 3500800 PMC2491032

[30] Zhang Z: Model building strategy for logistic regression: purposeful selection. *Annals of translational medicine* 2016, 4(6). doi: 10.21037/atm.2016.02.15 27127764 PMC4828741

[31] Destaye Shiferaw, Haimanot Gebrehiwot Moges: Risk factors for active trachoma among children aged 1‑9 years in Maksegnit town, Gondar Zuria District, Northwest Ethiopia. *Saudi J Health Sci* 2013, 2(3):202–206.

[32] Tadesse B, Worku A, Kumie A, Yimer SA: Effect of water, sanitation and hygiene interventions on active trachoma in North and South Wollo zones of Amhara Region, Ethiopia: A Quasi-experimental study. *PLoS neglected tropical diseases* 2017, 11(11):e0006080. doi: 10.1371/journal.pntd.0006080 29125849 PMC5699846

[33] Mengistu Kassahun, Shegaze Mulugeta, Woldemichael Kifle, Gesesew Hailay, Yohannes Markos: Prevalence and factors associated with trachoma among children aged 1–9 years in Zala district, gamo gofa Zone, southern ethiopia. *Clinical Ophthalmology* 2016, 10:1663–1670. doi: 10.2147/OPTH.S107619 27621585 PMC5010175

[34] Kemal Kassim, Kassim Jeylan, Aman Rameto, Abduku Mohammedawel, Tegegne Mekonnen, Biniyam Sahiledengle: Prevalence of active trachoma and associated risk factors among children of the pastoralist population in Madda Walabu rural district, Southeast Ethiopia: a community-based cross-sectional study. *BMC infectious diseases* 2019, 19(1):1–7. 31035947 10.1186/s12879-019-3992-5 PMC6489250

[35] WoldeKidan E, Daka D, Legesse D, Laelago T, Betebo B: Prevalence of active trachoma and associated factors among children aged 1 to 9 years in rural communities of Lemo district, southern Ethiopia: community based cross sectional study. *BMC Infectious Diseases* *volume* 2019, 19(1):1–8. 31651236 10.1186/s12879-019-4495-0 PMC6813116

[36] Emma M Harding-Esch Julbert Kadimpeul, Sarr Boubacar, Sane Awa, Badji Souleymane, Sillah Ansumana, et al: Population-based prevalence survey of follicular trachoma and trachomatous trichiasis in the Casamance region of Senegal. *BMC public health* 2018, 18(1):62. 10.1186/s12889-017-4605-0 PMC5530574 28747198

[37] Ngondi Jeremiah, Gebre Teshome, Shargie Estifanos B., Adamu Liknaw, Ejigsemahu Yeshewamebrat, Teferi Tesfaye, et al: Evaluation of three years of the SAFE strategy (Surgery, Antibiotics, Facial cleanliness and Environmental improvement) for trachoma control in five districts of Ethiopia hyperendemic for trachoma. *Transactions of the Royal Society of Tropical Medicine and Hygiene* 2009, 103(10):1001–1010. doi: 10.1016/j.trstmh.2008.11.023 19178920

[38] Nash Scott D, Stewart Aisha E P, Astale Tigist, Sata Eshetu, Zerihun Mulat, Gessese Demelash, et al: Trachoma prevalence remains below threshold in five districts after stopping mass drug administration: results of five surveillance surveys within a hyperendemic setting in Amhara, Ethiopia. *538–45* 2018, 112(12):538–545. doi: 10.1093/trstmh/try096 30265355 PMC6255692

[39] Solomon Anthony W, World Health Organization, International Trachoma Initiative: Trachoma control a guid for programme managers. 2015.

[40] World Health Organization: Validation of elimination of trachoma as a public health problem. In., Dr. Solomon A.edn: World Health Organization; 2016.

[41] Emma M. Harding-Esch, Tansy Edwards, Harran Mkocha, Beatriz Munoz, Holland Martin J., Burr Sarah E., et al: Trachoma Prevalence and Associated Risk Factors in The Gambia and Tanzania: Baseline Results of a Cluster Randomised Controlled Trial. *PLoS neglected tropical diseases* 2010, 4(11). doi: 10.1371/journal.pntd.0000861 21072224 PMC2970530

[42] Edwards Tansy, Harding-Esch Emma M., Hailu Girum, Andreason Aura, Mabey David C., Todd Jim, et al: Risk factors for active trachoma andChlamydia trachomatis infection in rural Ethiopia after mass treatment with azithromycin. *Tropical medicine & international health* 2008, 13(4):556–565. 18282237 10.1111/j.1365-3156.2008.02034.x

[43] Harding-Esch EM, Edwards T, Sillah A, Sarr-Sissoho I, Aryee EA: Risk factors for active trachoma in The Gambia. *Transactions of the Royal Society of Tropical Medicine and Hygiene* 2008, 102(12):1255–1262. doi: 10.1016/j.trstmh.2008.04.022 18502459 PMC3836170

[44] Zack R, Mkocha H, Zack E, Munoz B, West SK: Issues in defining and measuring facial cleanliness for national trachoma control programs. Trans R Soc Trop Med Hyg 102: 426–431. *Transactions of the Royal Society of Tropical Medicine and Hygiene* 2008, 102(5):426–431. doi: 10.1016/j.trstmh.2008.02.001 18346769

[45] Ngondi J, Gebre T, Shargie EB, Graves PM, Ejigsemahu Y, Teferi T, et al: Risk factors for active trachoma in children and trichiasis in adults: a household survey in Amhara Regional State, Ethiopia. *Transactions of the Royal Society of Tropical Medicine and Hygiene* 2008, 102(5):432–438. doi: 10.1016/j.trstmh.2008.02.014 18394663

[46] Golovaty Ilya, Jones Larissa, Gelaye Bizu, Tilahun Melkie, Belete Habtamu, Kumie Abera, et al: Access to water source, latrine facilities and other risk factors of active trachoma in Ankober, Ethiopia. *PLoS One* 2009, 4(8):e6702. doi: 10.1371/journal.pone.0006702 19693271 PMC2724741

[47] Ketema Kassahun, Tiruneh Moges, Woldeyohannes Desalegn, Muluye Dagnachew: Active trachoma and associated risk factors among children in Baso Liben District of East Gojjam, Ethiopia. *BMC public health* 2012, 12(1):1–7. doi: 10.1186/1471-2458-12-1105 23259854 PMC3543160

[48] Ngondi J, Matthews F, Reacher M, Onsarigo A, Matende I, Baba S, et al: Prevalence of risk factors and severity of active trachoma in southern Sudan: an ordinal analysis *The American journal of tropical medicine and hygiene* 2007, 77(1):126–132. 17620643

[49] Nigusie A, Berhe R, Gedefaw M: Prevalence and associated factors of active trachoma among children aged 1–9 years in rural communities of Gonji Kolella district, West Gojjam Zone, North West Ethiopia. *BMC research notes* 2015, 8(1):1–9. doi: 10.1186/s13104-015-1529-6 26530131 PMC4632356

