# Supplementary material for: Prevalence of active trachoma and its associated factors among 1–9 years of age children from model and non-model kebeles in Dangila district, northwest Ethiopia
Source: PLoS One. 2022 Jun 15;17(6):e0268441. doi: 10.1371/journal.pone.0268441 (PMC9200339; doi:10.1371/journal.pone.0268441)
Supplement: S1 File — (DOCX) [file pone.0268441.s001.docx]

**የስምምነትመግለጫቅፅ፤በአማርኛየተተረጎመ**

እንደምንአረፈዳችሁ/ዋላችሁ፡-ስሜ____________ይባላል፡፡የመጣሁትከደብረማረቆስዩኒቨረስቲበማህበረሰብጤናየ2ኛድግሪተማሪየሆነዉንአልማውገነትወክየመረጃለመሰብሰብነዉ፡፡

እኔ ጥናቱ ለ2ኛ ድግሪ ማሟያ እንደሆነና በደብረማርቆስ ዩኒቨርሲቲ ድጋፍ ተደርጎለት የሚሰራ እና አቶ አልማዉ ገነት የጥናቱ ተመራማሪ እንደሆነ አውቃለሁ፡፡ የጥናቱም አላማ በሚገባኝ ቋንቋ በመረጃ ሰብሳቢው የተገለፀልኝ ሲሆን አላማውም ከ1-9 አመት በሆኑ ህፃናት ላይ የሚከሰተውን የአፍላ ትራኮማ በሽታ ስርጭት ና ወሳኝ መንስኤዎችን መለየት ነው፡፡ እኔ የምሰጠው መረጃም በጥንቃቄ እንደሚያዝና ለሌላ ሰው ተላልፎ እንደማይሰጥ ተነግሮኛል፡፡ ጥናቱ በኔም ሆነ በልጄ ምንም አይነት ጉዳት እንደማያደርስና ምንም የአጭር ግዜ ጥቅምም ሆነ ማካካሻ እንደሌለው አምኛለሁ፡፡ መረጃውን በምሰጥበት ጊዜ የማልፈልገውን ጥያቄ መልስ ያለመስጠት ወይም ሙሉ በሙሉ መጠይቁን የማቋረጥ ሙሉ መብት እንዳለኝ ተነግሮኛል፡፡ ስለጥናቱ ያልገባኝን ነገር ቢኖር መረጃው ከመሰብሰቡ በፊትም ሆነ በሌላ ጊዜ ከዚህ በታች የተጠቀሱትን አድራሻ በመጠቀም መጠየቅ እንደምችል ተነግሮኛል፡፡

1. ደብረማርቆስ ዩኒቨርሲቲ; የቢሮ ስሌክ ቁጥር፡- ______________________
2. የጥናቱ ተመራማሪ ሙለ ስም እና አድራሻ፡- አልማዉ ገነት

ስሌክቁጥር፡ +ስሌክቁጥር፡ +251 911592228/0925183131 E-mail: yeshiwas690@gmail.com

የመረጃሰብሳቢተቆጣጣሪሙለስም _____________አድራሻ _________________

ከዚህም በላይ ያለውን ቅፅ አንብቢያለው ወይም በሚገባኝ ቋንቋ ተነቦልኝ የምጠየቀዉን ጥያቄ ለመመለ ስፍቃደኛ ሆኜ ተስማምቻለው፡፡

አዎፈ ቃደኛ ነኝ ፊርማ __________ ፈቃደኛ አይደለሁም ፊርማ

(ለመሳተፍፈ ቃደኛ ካልሆኑ አመስግነዉ ይለፉ)

የመረጃ ሰብሳቢ ስም ________________ ፊርማ ____________ ቀን_____________

- 1. የአማርኛመጠይቅ

የተሳታፊመለያቁጥር______ ቀበሌ______የቃለ መጠይቅ አድራጊው ስም_______ ቀን____ የተጀመረበ ሰዓት___________የተጠናቀቀበት ሰዓት ________________

**ክፍል 1፡የሶሽዮ-ዲሞግራፉመረጃመጠይቅ**

| **ተ/ቁ** | **ጥያቄ** | **አማራጭ** | **ኮድ** | **እለፍ** |
| --- | --- | --- | --- | --- |
| 101 | የሕፃኑ/ኗእናት/አሳዳጊዕድሜበአመት | _________________ |  |  |
| 102 | የሕፃኑ/ኗዕድሜ | _________________ |  |  |
| 103 | የሕፃኑ/ኗፆታ | ወንድ  ሴት | 1  2 |  |
| 104 | የህፃኑ/ኗ የትምህርት ደረጃ | ትምህርት ያልጀመረ  አንደኛ ደረጃ | 1  2 |  |
| 105 | የቤተሰብወይምያአሳዳጊሀይማኖት | ኦርቶዶክስ  ሙስሊም  ፕሮቴስታንት  ካቶሊክ  ሌላካለይገለፅ______ | 1  2  3  4  99 |  |
| 106 | የሕፃኑ/ኗእናት/አሳዳጊየትምህርትደረጃ | መደበኛ ትምህርት ያልተከታተሉ  አንደኛ ደረጃ (1-8)  ሁለተኛደረጃ (9-12)  ኮሌጂእናዩንቨርስቲ | 1  2  3  4 |  |
| 107 | የሕፃኑ/ኗእናት/አሳዳጊየስራሁኔታ | ስራየሌላት  የመንግስትሰራተኛ  ነጋዴ  ገበሬ  ሌላካለይገለፅ______ | 1  2  3  4  99 |  |
| 108 | የሕፃኑ/ኗእናት/አሳዳጊየጋብቻሁኔታ | ያላገባች  ያገባች  የፈታች  ባልየሞተባት  የተለያዩ(ተጋብተው በተለያየ ቦታ የሚኖሩ) | 1  2  3  4  5 |  |
| 109 | የባለቤትዎየትምህርትደረጃ | መደበኛ ትምህርት ያልተከታተሉ  አንደኛ ደረጃ (1-8)  ሁለተኛደረጃ (9-12)  ኮሌጂእናዩንቨርስቲ | 1  2  3  4 |  |
| 110 | የባለቤትዎየስራሁኔታ | ስራየሌለው  የመንግስትሰራተኛ  ነጋዴ  ገበሬ  ሌላካለይገለፅ_____ | 1  2  3  4  99 |  |
| 111 | ጠቅላላ የቤተሰብ ቁጥር ብዛት | ________________ |  |  |
| 112 | ጠቅላላ ከ10 አመት በታች ህፃናት ብዛት | ________________ |  |  |
| 113 | የመኝታ ክፍሎችን ወይም የመኝታ አልጋዎችን በጋራ ይጠቀማሉ | አዎን  የለም | 1  0 |  |
| 114 | ከህፃኑ/ከህፃኗ ጋር ያለወት ዝምድና | የስጋ ወላጅ  የህፃኑ ተንከባካቢ | 1  2 |  |
| 115 | የመኖሪያ ቦታ | ከተማ ገጠር | 1  2 |  |

## የሀብትሁኔታጠቋሚመረጃ

| ተ/ቁ | ጥያቄ | አማራጭ | ኮዴ | እሇፍ |
| --- | --- | --- | --- | --- |
| 116 | የት ነው የሚኖሩት? | ከራሳችን ቤት  ከኪራይ ቤት | 1  2 |  |
| 117 | የሚኖሩበት ቤት ስንት ክፍልች አለት? | ________________ |  |  |
| 118 | የቤቱ ግዴግዲ የተሰራው ከምን ነው? በእይታ ይሞላ | ከእንጨትሁኖጭቃየሌለዉ  ከእንጨትእናከጭቃ  ከእንጨትእናከሲሚነቶ  ከብሎኬትየተሰራ  ሌላካለይጥቀሱ_________ | 1  2  3  4  99 |  |
| 119 | የቤቱ ጣሪያ የተሰራው ከምን ነው? በእይታ ይሞላ | ከሳር/ከቅጠሌ  ከቆርቆሮ | 1  2 |  |
| 120 | ቤቱ ስንት ምኝታ ክፍሌ አለዉ? | ________________ |  |  |
| 121 | የቤቱ ወለል የተሰራው ከምን ነው? በእይታ ይሞላ | የተፈጥሮመሬት /ወለልምንምነገርየሌለዉ  በከብቶችእበትየተሰራ /የተለቀለቀወለል  በእንጨትየተሰራወለል  በሲሚነቶየተሰራወለል  ሌላካለይጥቀሱ_________ | 1  2  3  4  99 |  |
| 122 | ቤት ውስጥ ለማብሰያነት የሚጠቀሙት የሃይሌ ምንጭ ምንዴን ነው? | እንጨት/ኩበት  ከሰል  ኤላክትሪክ  ጋዝ  ሌላካለይጥቀሱ_________ | 1  2  3  4  99 |  |
| 123 | በቤት ውስጥ የሚጠቀሙትየብርሃን ምንጭ ምንዴን ነው? | ነጭ ጋዝ  ኤላክትሪክ  ሶሊር  ሻማ  ሌላካለይጥቀሱ_________ | 1  2  3  4  99 |  |
| 124 | የቤተሰብዎ አባሊት በዋናነት የሚጠቀመው የውኃ መገኛ የትኛው ነው? | በግቢ ውስጥ ያለ የቧንቧ ውኃ  ከጎረቤት ያለ የቧንቧ ውኃ  በእጅ የሚነቀነቅ የጋራ ዉሃ  ቦኖ ዉሃ  የተከለለ የጉዴጋዴ /የምንጭ ዉሃ  የዝናብ ውኃ  ያልተከለለ የጉዴጋዴ/የምንጭ ዉሃ  የምንጭ /የወራጅ/ ኩሬ የግዴብ ዉሃ  ሌላካለይጥቀሱ_________ | 1  2  3  4  5  6  7  8  99 |  |
| 125 | ለቤተሰብዎ የሚያገለግል የመፀዳጃ ቤት አለ | አዎን  የለም | 1  0 |  |
| 126 | የመጸዲጃ ቤቱ ምን አይነት አንዯሆነ በምሌከታ ይረጋገጥ | በዉሃ የሚሰራ ሽንት ቤት  የአየር ማስወጫ ቱቦ ያለዉ ሽንት ቤት  ርብራብ የሌለው ባህሊዊ ሽንት ቤት  ርብራብ ያለው ባህሊዊ ሽንት ቤት  ሽንት ቤት የለም/ቁጥቋጦ/ሜዲ  ሌላካለይጥቀሱ_________ | 1  2  3  4  5  99 |  |
| 127 | ቤተሰቡመፀዳጃ ቤቱንይጠቀሙበታል | አዎን  የለም | 1  0 |  |
|  | ክዚህ በታች ከተዘረዘሩት ንብረቶች በቤታችሁ ዉስጥ ያሊችሁ የቱ ነዉ? (ከአንዴ በሊይ መመሇስ ይቻሊሌ) | አማራጭ | ኮዴ | እሇፍ |
| 128 | ሬድዮ | አዎን  የለም | 1  0 |  |
| 129 | ቴሌቪዥን | አዎን  የለም | 1  0 |  |
| 130 | የቤትስልክ | አዎን  የለም | 1  0 |  |
| 131 | ፍሪጅ | አዎን  የለም | 1  0 |  |
| 132 | ወንበር | አዎን  የለም | 1  0 |  |
| 133 | ጠረንጴዛ | አዎን  የለም | 1  0 |  |
| 134 | የጥጥ/የእስቦንጅ / አስፕሪነግፍራሽያለዉአልጋ | አዎን  የለም | 1  0 |  |
| 135 | ሞባይልስልክ | አዎን  የለም | 1  0 |  |
| 136 | ሳይክል | አዎን  የለም | 1  0 |  |
| 137 | ሞተርሳይክል | አዎን  የለም | 1  0 |  |
| 138 | የፈረስጋሪ | አዎን  የለም | 1  0 |  |
| 139 | ባጃጅ/መኪና | አዎን  የለም | 1  0 |  |
| 140 | የባንክቡክ | አዎን  የለም | 1  0 |  |
| 141 | ሰአት | አዎን  የለም | 1  0 |  |
| 142 | ሶፋ | አዎን  የለም | 1  0 |  |
| 143 | ላሊ ካለ ይጥቀሱ _________________________ |  | 99 |  |
| 144 | የግላችሁየሆነለምርት/እርሻየሚሆንመሬትአላችሁወይ ? | አዎን  የለም | 1  0 |  |
| 145 | ለጥያቄቁጥር 142 የተሰጠውመልስአዎንከሆነየመሬቱ ብዛትምንያህሌይሆናሌ? | ____________ ሄክታር /1ሄ = 4 ጥማዴ/ |  |  |
| 145 | ከሚከተለት የቤት እንሰሳት ዉሰጥ የትኛው አሊችሁ? | አማራጭ | ኮዴ |  |
| 146 | በሬ፣ላም | አዎን  የለም | 1  0 |  |
| 147 | ፈረስ/አህያ፣በቅሎ | አዎን  የለም | 1  0 |  |
| 148 | ፍየል | አዎን  የለም | 1  0 |  |
| 149 | በግ | አዎን  የለም | 1  0 |  |
| 150 | ዶሮ | አዎን  የለም | 1  0 |  |
| 151 | የንብቀፎ | አዎን  የለም | 1  0 |  |
| 152 | ሌላካለይጥቀሱ ______________________________ |  | 99 |  |

ክፍል**2:** የቤትእናየአካባቢሁኔታ

| ተ/ቁ | ጥያቄዎች |  | አማራጭ | ኮዴ | እሇፍ |
| --- | --- | --- | --- | --- | --- |
| 201 | የቤት እንስሳት አላችሁ ወይ? |  | አወን  የለም | 1  0 |  |
| 202 | የቤት እንስሳት ካሎችሁ የት ነዉ የሚያዴሩት? |  | ዉጭላይ/በረትላይ  የተለየክፍልማደሪያአላቸዉ  ከቤተሰቡጋርበአንድክፍል | 1  2  3 |  |
| 203 | በመኖሪያ ቤቱ ዙሪያ የሚታይ የእንስሳት ፅዲጅ አለ ወይ? |  | አወን  የለም | 1  0 |  |
| 204 | በመኖሪያ ቤቱ ዙሪያ የሚታዩ ዝንቦች አለ ወይ? |  | አወን  የለም | 1  0 |  |
| 205 | በመኖሪያ ቤቱ ውስጥ የሚታዩ ዝንቦች አለ ወይ? |  | አወን  የለም | 1  0 |  |
|  | የውኃ አቅርቦትን በተመሇከተ | አማራጭ |  | ኮዴ | እሇፍ |
| 206 | ብዙ ጊዜ ውኃ የሚቀዱት በምንድን ነው? |  | 10 ሊትር በሚይዝ ጀሪካን/ማሰሮ  15 ሊትር በሚይዝ ጀሪካን/ማሰሮ  20 ሊትር በሚይዝ ጀሪካን/ማሰሮ  25 ሊትር በሚይዝ ጀሪካን/ማሰሮ  ሌላ ካለ ይገለጽ________________ | 1  2  3  4  99 |  |
| 207 | በዚህ የውኃ መቅጃ እቃ ለሁሉም አይነት አገልግሎት በቀን ምን ያህል ጊዜ ይቀዳሉ? | **____________**ጀሪካን/ማሰሮ |  |  |  |
| 208 | ቤተስብዎ በቀን በአማካይ ምን ያህሌ ሉትር ውኃ ይጠቀማሌ? |  | _________________ |  |  |
| 209 | የሚጠቀሙትን ውኃ ወደ መገኛው ሂድ ቀዴቶ ለመመለስ ምን ያህሌ ጊዜ ይጨርሳሌ? |  | የውኃ መገኛው በግቢ ውስጥ ነው  < 30 ደቂቃ ይጨርሳሌ  > 30 ደቂቃ ይጨርሳሌ | 1  2  3 |  |
|  | የመጸዲጃ ቤትን በተመአለከተ | አማራጭ |  | ኮዴ | እለፍ |
| 210 | እርስዎና ጎሌማሳ የሆኑ የቤተሰብዎ አባላት የምትጸዲደት የት ነው? | በግሌ መጸዲጃ ቤት  በጋራ መጸዲጃ ቤት  መጸዲጃ ቤት የለም ፣ ውጭ ቤት አጠገብ  መጸዲጃ ቤት የአለም፣ቁጥቋጦ ውስጥ/ ሜዲ ላይ  ሌላካለይጥቀሱ_________ | | 1  2  3  4  99 |  |
| 211 | በመኖሪያ ቤቱ ዙሪያ የሰዎች ፅዲጅ መኖሩ በምሌከታ ይረጋገጥ | አወን  የለም | | 1  0 |  |
| 212 | ከመኖሪያ ቤትዎ የሚጠረውን የደረቅ ቆሻሻ በየትኛው መንገዴ ያሰወግዲለ? | ቤቱ አቅራቢያ ውጭ ሊይ ወይም ሜዲ ሊይ በመወርወር  በተዘጋጀ የደረቅ ቆሻሻ ጉድጓድግቢ ውስጥ በማቃጠሌ በመዘጋጃ ቤት ይሰበሰባሌ  ሌላካለይጥቀሱ_________ | | 1  2  3  99 |  |
| 213 | ከዋናው መኖሪያ ቤት በ 20 ሜትር ርቀት ሊይ የደረቅ ቆሻሻ ጉድጓድመኖሩ ይረጋገጥ | አለ  የለም | | 1  0 |  |
| 214 | የፍሳ ቆሻሻማስወገጃጉድጓድመኖሪያ ቤትአቅራቢያ አለ | አለ  የለም | | 1  0 |  |

**ክፍል 3፡- የህፃናት የንፅህና እና የአፍላ ትራኮማ ሁኔታ**

| **ተ/ቁ** | **ጥያቄ** | **አማራጭ** | **ኮድ** | **እለፍ** |
| --- | --- | --- | --- | --- |
| 301 | የልጆችዎን ፊት ዘወትር ያጥባሉ | አወን  የለም | 1  0 |  |
| 302 | ለጥያቄቁጥር 142 የተሰጠውመልስአዎንየልጆችዎን ፊት በቀን ምን ያህል ጊዜ ያጥባሉ | አንድ ጊዜ  ሁለት ጊዜ  ከሁለት ጊዜ በላይ  አላወቀውም | 1  2  3  4 |  |
| 303 | የልጆችዎን ፊት በሚያጥቡበት ወቅት ሳሙና የጠቀማሉ | አወን ዘወትር/ሁልጊዜ  አወን አልፎአልፎ  አልጠቀምም | 1  2  3 |  |
| 304 | የልጆችዎን ፊት ካጠቡ በኃላ በፎጣ ያደርቃሉ? | አወን፣ ዘወትር  አወን፣ አልፎአልፎ  አልጠቀምም | 1  2  3 |  |
| የህፃኑ/ኗ የፊት ንጽህና ሁኔታ ምን የመስላል በምልከታ | | ይረጋገጥ |  |  |
| 305 | ከህፃኑ/ኗ አይን የሚወጣ ፈሳሽ | 0አለ  የለም | 1  0 |  |
| 306 | ከህፃኑ/ኗ አፍንጫየሚወጣ ፈሳሽ | አለ  የለም | 1  0 |  |
| 307 | ከህፃኑ/ኗ ፊት ዝንቦች | አለ  የለም | 1  0 |  |
| 308 | ከህፃኑ/ኗ አይን ዝንብ | አለ  የለም | 1  0 |  |
| 309 | ከህፃኑ/ኗ አይን ላይ ቅምጥጥ አለ | አለ  የለም | 1  0 |  |
| የህፃኑ/ኗ አይን የአፍላ ትራኮማ ልየታ ውጤት | |  |  |  |
| 310 | የህፃኑ/ኗ አይን የላይኛው ቆብ ላይ የአፍላ ትራኮማ ምልከት አለ ወይ? | የአፍላ ትራኮማ ምልከትየለውም  የአፍላ ትራኮማ ምልከትአለ  የአፍላ ትራኮማ ለመለየት ያስቸግራል | 0  1  2 |  |
| 311 | ከህፃና/ ከህፃኑ የተገኘዉ የአፍላ ትራኮማ ትራኮማ አይነት | TF  TI | 1  2 |  |

**ክፍል 4፡- የአፍላ ትራኮማ ብክለትን ለመከላከል እየተሰሩ ያሉ ስራዎች** ና **ሰለትራኮማ በሽታ ያላቸው እውቀት**

| **ተ/ቁ** | **ጥያቄ** | **አማራጭ** | **ኮድ** | **እለፍ** |
| --- | --- | --- | --- | --- |
| 401 | የአፍላትራኮማልየታየተደረገለትህፃንዎየአፍላትራኮማመከላከያመድኃኒትስንት ጊዜ ወስዷል? | ወስዶአያውቅም  አንድጊዜ  ሁለትጊዜ  ሶስትጊዜ  አራትጊዜእናከዚያበላይ | 1  2  3  4  5 |  |
| 402 | የአፍላትራኮማልየታየተደረገለትህፃንዎየአፍላትራኮማመከላከያመድኃኒት ወስዶ/ዳ የሚያውቅ ከሆነ በስንት ጊዜ ልዩነት ነው የወሰደው/ችው? | በአመት ሁለት ጊዜ  በአመት አንድ ጊዜ  በሁለት አመት አንድ ጊዜ | 1  2  3 |  |
| 403 | የአፍላትራኮማልየታየተደረገለትህፃንዎየአፍላትራኮማመከላከያመድኃኒት ወስዶ/ዳ የሚያውቅ ከሆነ ለመጨረሻ ጊዜ የወሰደው/ችው መቸ ነው? | ከሶስት ወር በፊት  ከስድስት ወር በፊት  ከዘጠኝ ወር በፊት  ከአንድ አመት በፊት  ከሁለት በፊት | 1  2  3  4  5 |  |
| ስለትራኮማበሽታየተሰጠየጤናትምህርት | | |  |  |
| 404 | ስለትራኮማበሽታምንነትየጤናትምህርትአግኘተውያውቃሉ? | አወን  የለም | 1  0 |  |
| 405 | ለጥያቄቁጥር 404መልስዎአዎንከሆነ፣የጤናትምህርቱየተሰጠውበማንነው? | በጤናባለሙያ  በጤናኤክሰቴንሽንባለሙያ በሚዲያ/በራዲዮ/በቴሌቭዥን  ከአቻዎቸ/ከጎረቤቶቸ  ሌላካለይገለፅ________________ | 1  2  3  4  99 |  |

| **406** | የትራኮማበሽታምንምንምልክቶችአሉት? | ምንምምልክትየለውም  ማቃጠል  ማሳከክ የብርሃንጨረርንመፍራት  ከአይንየሚወጣፈሳሽነገርመኖር  ወደአይንባዕድነገርአንደገባአይነትሰሜት/መቆርቆር  የአይንመቅላት  አላውቅም | 1  2  3  4  5  6  7  8 |  |
| --- | --- | --- | --- | --- |
| **407** | ሰዎችየትራኮማበሽታንከየትያገኙታል? | ከታመሙሰዎቸች  ከእንስሳት  ከቆሻሻ  ሌላካለይገለፅ__ | 1  2  3  99 |  |
| **408** | የትራኮማበሽታከሰውወደሰውእንዴትይተላለፋል? | በዝንቦችአማካኝነት  የፊትማድረቂያፎጣንበጋራበመጠቀም  የአልጋልብሶችንበጋራበመጠቀም  የአይንመዋቢያአቃዎችንበጋራበመጠቀም  በሰውነትንክኪ  ንፅህናውባለተጠበቀእጅአይንንበመንካት  አላውቅም | 1  2  3  4  5  6  7 |  |
| **409** | የተራኮማበሽታንእንዴትመከላከልይቻላል? | መድኃኒትወይምከኒንበመውሰድ  የግልንፅሀናንበመጠበቅ  ሁለጊዜመጸዳጃቤትበመጠቀም  የአካባቢያችንንንጽህናበመጠበቅ  አላውቅም  ሌላካለይገለፅ________________ | 1  2  3  4  5  99 |  |
