# Supplementary material for: Prevalence of active trachoma and its associated factors among 1–9 years of age children from model and non-model kebeles in Dangila district, northwest Ethiopia
Source: PLoS One. 2022 Jun 15;17(6):e0268441. doi: 10.1371/journal.pone.0268441 (PMC9200339; doi:10.1371/journal.pone.0268441)
Supplement: S2 File — (DOCX) [file pone.0268441.s002.docx]

**Informed consent agreement form, English version**

Good morning/afternoon. My name is ______________and I am from Debremarkos University, I am a member of a data collector team on behalf the study conducted by Almaw Genet, who is a MPH. student in Debremarkos University.

prevalence and associated factors of active trachoma among children 1-9 years’ age from model and non-model kebeles in Dangila woreda, Northwest Ethiopia, 2019.

I am aware that this research undertaking is a post graduate MPH. degree research project which is fully supported and coordinated by Debremarkos University and the designate principal investigator is Almaw Genet.

I have been also fully informed in the language I understood and about the research project objective to assess the prevalence and associated factors of active trachoma among children 1-9 years’ age in model and non-model kebeles in Dangila woreda, Northwest Ethiopia, 2019.

I have been informed that all the information I shall provide to the interviewer will be kept confidential. I understood that the research has no any risk and no compensation. I also know that I have the right to withhold information, skip questions to answer or to withdraw from the study any time. I have been informed that nobody will impose on me to explain the reason of withdrawal. It is also clear that there will be no effect at all in my health benefit or other administrative effect that I get from the district. I have been assured of the right to ask information that is not clear about the research before and/or during the research work by contacting:

1. Debremarkos University, Office phone:

2. Principal investigator name and address: **Almaw Genet, cell Phone: +251 (0) 911592228**

3. Supervisor name and address: _____________________

I have read this form, or it has been read to me in the language I comprehend, and I understood the condition stated above; therefore, I am willing and confirm my participation by signing this consent form. Mothers/caregivers agreed to participate in the study: (Mark one of them for verbal/oral consent) Yes __________ No __________

Name of interviewer signature ________________

Signature __________________Date ____________________

- 1. - **English Questionnaire**

Participant ID No___________ Kebele ____ Interviewer name_________________________ Date of interview____________ Starting time _____________Ending time ___________

Please encircle the correct answer which to indicate number and write a correct number example age put in a year.

**Part 1: Socio-demographic characteristics**

| S.No | Questions | Answer | Code | Skip |
| --- | --- | --- | --- | --- |
| 101 | Age of the mother/caregiver(years) | -------------------- |  |  |
| 102 | Age of the child | -------------------- |  |  |
| 103 | Sex of child | Male  Female | 1  2 |  |
| 104 | Level ofeducation of a child | None  Primary | 1  2 |  |
| 105 | Religion of mother/caregiver | Orthodox  Muslim  protestant  catholic  Others----- | 1  2  3  4  99 |  |
| 106 | Level of education of mother/caregiver | Not attended formal education  Primary level (1-8)  Secondary level (9-12)  Diploma and above | 1  2  3  4  5 |  |
| 107 | Maternal occupation | Unemployed  Civil servant  Merchant  Farmer  Other_______ | 1  2  3  4  99 |  |
| 108 | Marital status of mother/caregiver | Single  Married  Divorced  Widowed  separated | 1  2  3  4  5 |  |
| 109 | what is the educational level of your husband? | Not attended formal education  Primary level (1-8)  Secondary level (9-12)  Diploma and above | 1  2  3  4  5 |  |
| 110 | what is occupational status of your husband? | Unemployed  Civil servant  Merchant  Farmer  Other_______ | 1  2  3  4  99 |  |
| 111 | Total number of family size | _______________ |  |  |
| 112 | Total number of <10 years children | _______________ |  |  |
| 113 | Do children share same sleeping space or bedding? | Yes  No | 1  0 |  |
| 114 | What is your relationship with the children? | Parent  Guardian | 1  2 |  |
| 115 | Residence | Urban  Rural | 1  2 |  |

**Wealth index (Ownership of durable assets and housing characteristics)**

| 116 | Where do you live? | Own house  Rented house | 1  2 |  |
| --- | --- | --- | --- | --- |
| 117 | Number of rooms in the dwelling place | ________________ |  |  |
| 118 | What is the wall of the house-made off?  check by observation | Wood but not have a mod  Wood with mod  Wood and cement  Blocket  Others specify_________________ | 1  2  3  4  99 |  |
| 119 | Observe which material the house roof is made off? | Grass/ leaf corrugated iron | 1  2 |  |
| 120 | How many bedrooms in the house | ________________ |  |  |
| 121 | What is the floor of the house-made off?  check by observation | Natural ground  Muck/smooth by cow’s faces  Wood  Cement  Other Specify)_____________ | 1  2  3  4  99 |  |
| 122 | What is your main source of cooking fuel? | Firewood /Animal dung  Charcoal  Electricity  Kerosene/gas  Others Specify___________ | 1  2  3  4  99 |  |
| 123 | What are the main sources of lighting? | Kerosene  Electricity  Solar  Candle  Others specify_________________ | 1  2  3  4  99 |  |
| 124 | Where do you get drinking water? | Piped into the yard/plot water  Piped to neighbor  Hand pumps  Public tap/standpipe  Protected well/spring  Rainwater  Unprotected well/spring  Surface water (river/dam/ /pond/stream/canal/ irrigation channel)  Other list____________________ | 1  2  3  4  5  6  7  8  99 |  |
| 125 | Do you have a latrine for the household members? | Yes  No | 1  0 |  |
| 126 | Observe which type of latrine the household has? | pour-flush latrine  Ventilated improved pit latrine (VIP)  Pit latrine with slab  Pit latrine without slab/open pit  No facilities or bush or field  Other (specify )_________________ | 1  2  3  4  5  99 |  |
| 127 | Does the family utilize the latrine? Observation | Yes  No | 1  0 |  |
|  | Among the following materials, which one do you own? (more than one answer is possible ) |  |  |  |
| 128 | Radio | Yes  No | 1  0 |  |
| 129 | Television | Yes  No | 1  0 |  |
| 130 | House phone | Yes  No | 1  0 |  |
| 131 | Fridge | Yes  No | 1  0 |  |
| 132 | Chair | Yes  No | 1  0 |  |
| 133 | Table | Yes  No | 1  0 |  |
| 134 | Bed and mattress which made from cotton spring | Yes  No | 1  0 |  |
| 135 | Mobile | Yes  No | 1  0 |  |
| 136 | Cycle | Yes  No | 1  0 |  |
| 137 | Motor cycle | Yes  No | 1  0 |  |
| 138 | Horse’s cart | Yes  No | 1  0 |  |
| 139 | Bajaj/car | Yes  No | 1  0 |  |
| 140 | Bank book | Yes  No | 1  0 |  |
| 141 | Watch | Yes  No | 1  0 |  |
| 142 | Sofa | Yes  No | 1  0 |  |
| 143 | If others (specify)_______________ |  | 99 |  |
| 144 | Do you have your own farm for the purpose of agriculture/cropping? | Yes  No | 1  0 |  |
| 145 | If the answer for Q#142 is 1, how many hectares? | ___________________ |  |  |
|  | From the following household animal do you have? (can answer more than one) |  |  | Number |
| 146 | Ox/ cow | Yes  No | 1  0 |  |
| 147 | Horse/donkey/ mule | Yes  No | 1  0 |  |
| 148 | Goat | Yes  No | 1  0 |  |
| 149 | Sheep | Yes  No | 1  0 |  |
| 150 | Hen | Yes  No | 1  0 |  |
| 151 | Beehive | Yes  No | 1  0 |  |
| 152 | Others(specify)_________________ |  | 99 |  |

**Part II: Household Level Environmental factors**

| **S/No** | **Questions** | **Answer** | Code | **skip** |
| --- | --- | --- | --- | --- |
| 201 | Do you have livestock? | Yes  No | 1  0 |  |
| 202 | If you have livestock, where do they live? | Outside the room/‟beret laye‟  Separate room for themselves  In the same room with family | 1  2  3 |  |
| 203 | Are there animal feces observed around the house? | Yes  No | 1  0 |  |
| 204 | Are there flies in the house? | Yes  No | 1  0 |  |
| 205 | Flies were observed around the house | Yes  No | 1  0 |  |
| 206 | What is your common water collection material? | 10 litrejerrycan/pot  15 litrejerrycan/pot  20 litrejerrycan/pot  25 litrejerrycan/pot  Other specify ___________________ | 1  2  3  4  99 |  |
| 207 | How many times do you collect water per day with this collection material? | **___________________** |  |  |
| 208 | On average, how much litter of water does your family use per day | **_____________________** |  |  |
| 209 | How long does it take to get water and come back? | Water source in the yard  ≤ 30 minutes  ≥30 minutes | 1  2  3 |  |
| 210 | Where do you and other adults in the household usually defecate? | Private latrine  Shared latrine  No structure outside near the house  No structure in the bush or field  Other(specify)______________ | 1  2  3  4  99 |  |
| 211 | Observe the presence of feces near the vicinity of the main house | Yes  No | 1  0 |  |
| 212 | What method do you use to dispose of the solid waste which is generated in the house? | Throwing near the house /open field/  In the pit prepared for solid waste  burning near the yard  collected by municipality  Other(specify)______________ | 1  2  3  4  99 |  |
| 213 | Is there evidence of solid waste or garbage within 20 meters of the house ( This does not include animal droppings) | Yes  No | 1  0 |  |
| 214 | Is there a liquid waste segregation pit is available | Yes  No | 1  0 |  |

**Part 3፡- Child’s behavioral factors and status of Active trachoma**

| **S.no** | **Questions** | **Answer** | **Code** | **Skip** |
| --- | --- | --- | --- | --- |
| 301 | Do you wash your face regularly? | Yes  No | 1  0 | Skip to 199 |
| 302 | If question 301 Ans is “yes,” how many times do you wash your face per day? | Once  Twice  More than twice  Don’t know | 1  2  3  4 |  |
| 303 | Do you use soap when you are washing your face? | Yes, regularly/always  Yes, sometimes  Never | 1  2  3 |  |
| 304 | Do you dry your face after washing with a towel? | Yes, regularly  Yes, sometimes  Never | 1  2  3 |  |
|  | **Facial cleanliness observation** |  |  |  |
| 305 | Is there an Ocular discharge? | Yes  No | 1  0 |  |
| 306 | Is there Nasal discharge? | Yes  No | 1  0 |  |
| 307 | Are there Flies on the face? | Yes  No | 1  0 |  |
| 308 | Are there Fly-eye contacts? | Yes  No | 1  0 |  |
| 309 | Is there Sleep in the eyes? | Yes  No | 1  0 |  |
|  | **Eye examination** |  |  |  |
| 310 | Is there a sign of active trachoma present in the eye? | Sign absent  Sign present  Not able to grade | 1  2  3 | Ans no Skip to 210 |
| 311 | Types of active trachoma observed by physical checkup | TF  TI | 1  2 |  |

**Part 4:- Interventions measures and Knowledge about trachoma**

| **S.no** | **Questions** | **Answer** | **Code** | **Skip** |
| --- | --- | --- | --- | --- |
| **401** | How many times has the child received azithromycin? | Not received yet  One time  Two times  Three times  Four and more times | 1  2  3  4  5 |  |
| **402** | If the child has received azithromycin, by what time interval did he/she get the drug? | Once a year  Two times a year  Every two years | 1  2  3 |  |
| **403** | If the child has received azithromycin when he/she gets the drug for the last time? | Three months before  Six months before  Nine months before  Before a year  Before two years | 1  2  3  4  5 |  |
|  | **Health Education about Trachoma** |  |  |  |
| 404 | Have you ever been informed about trachoma? | Yes  No | 1  0 | Ans is no skip to 213 |
| 405 | If question 404 Ans is “yes,” from which source do you hear? | Health personnel other than HEW  HEW Media/radio, TV  From peers, Community  Others/Specify___________ | 1  2  3  4  99 |  |
| 406 | What are the sign & symptoms of trachoma disease? | No Sign and Symptom  Burning  Itching Photophobia  Ocular discharge  Foreign body sensation  Red-eye  I don’t know | 1  2  3  4  5  6  7  8 |  |
| 407 | Where have people got trachoma from? | From diseased people  From animals  From dirt  Others/specify____________ | 1  2  3  99 |  |
| 408 | How is trachoma transmitted from person to person? | By flies  By sharing towels  By sharing bed sheets  By sharing eye makeup  By close contact of body  By touching eyes with unwashed hands I don’t know | 1  2  3  4  5  6  7 |  |
| 409 | How do we prevent trachoma? | Taking medicine/drugs  Keeping personal hygiene  Proper use of latrine  Making the compound clean  I don’t know  Others/specify____________ | 1  2  3  4  5  99 |  |
